# Development of the KeyStrokes test: An online neuropsychological assessment for attention, processing speed and executive function

**DOI:** 10.1111/jnp.12426

**Published:** 2025-05-03

**Authors:** Michael Lopez, John Fulton, Hayley Kristinsson, Sahra Kim, Elizabeth Stuart, Patrick Chen, Aaron Thomas, Megan Hussey‐Zommers, Rohan Roy, Arunima Kapoor, Alexis Conrad

**Affiliations:** ^1^ Department of Neurology University of California Irvine Health Irvine California USA; ^2^ Department of Pediatrics University of Utah School of Medicine Salt Lake City Utah USA; ^3^ Memory Gains Newton Massachusetts USA; ^4^ Miller Children’s Hospital Long Beach California USA; ^5^ Independent Researcher

**Keywords:** neuropsychology, online assessments, test development, test validity

## Abstract

The ‘KeyStrokes’ test (KS) was created as an online and computerized neuropsychological assessment to assess simple attention, processing speed, and executive function. This pilot study aims to show proof of concept of the KS test as a computerized assessment. Building on a previous feasibility study, we assessed the KS test's internal consistency and correlations to other neurocognitive assessments. Participants were recruited from a clinical sample of patients referred for standard neuropsychological evaluation and were asked to perform several standard neurocognitive tests and six subtests of the KS: two response time trials (arrows, words), three inhibition trials (arrows, words, arrows/words) and one inhibition/switching trial (arrows/words). We assessed internal consistency; conducted correlation analyses between each KS subtest, standard neuropsychological tests, and demographic characteristics (age, education, ethnicity, and gender); and conducted multiple regression analyses to assess the relationship between test performance and age and education. We assessed 87 individuals with a mean age of 54.09 years. Correlations between KS subtests were positive and strong (all above *ρ* > .72, *p* < .001). Subtests were generally positively correlated with select WAIS‐IV and Reynolds Interference Task subtests, and negatively correlated with trail making tests, the grooved pegboard test, and age. Age significantly predicted performance (*p* < .001), whereas education did not. Ethnicity appeared to correlate with certain subtests, whereas gender did not. Analysis of correlations between the KS subtests and multiple well‐established neuropsychological tests showed the possible viability of the KS as a new neurocognitive measure assessing areas of attention, processing speed, and executive function. Additional study of the KS can provide more evidence for its use as a new computerized, and possible online neuropsychological assessment.

## INTRODUCTION

Neuropsychology has been generally slow to adapt technology into clinical practice, including computerized tests and remote assessment, which impedes not only clinical practice but also the advancement and relevance of the field (Miller & Barr, 2017; Parsey & Schmitter‐Edgecombe, 2013). With advances in technology, there is a need to shift to using computerized neuropsychological measures, which are becoming a crucial component of clinical evaluations (Canini et al., 2014). In 1986, the American Psychological Association (APA) released guidelines for computerized assessments which noted several benefits including precise records of certain aspects of performance (e.g. latency, speed, errors), automated collection of data and storage, and overall efficiency (American Psychological Association, 1986; Schoenfeldt, 1989). Additionally, relative to paper and pencil methods, computer‐based tests allow for greater objectivity and standardization, can be tailored to the patient's ability, are easier to distribute (Burke & Normand, 1987), and can reduce floor and ceiling effects (Wild et al., 2008).

Despite benefits, computer‐based tests have limitations. Results from computer‐based assessments may not always be comparable to traditional forms of testing because of different experiences, particularly for speeded tests (Bauer et al., 2012; Mead & Drasgow, 1993; Schatz & Browndyke, 2002; Wojcik et al., 2019). For example, significant discrepancies between results on the computerized and the manual version of the Wisconsin Card Sorting Test (WCST) suggest that they are not psychometrically equivalent (Feldstein et al., 1999). Some computerized tests also do not have adequate psychometric properties (Wild et al., 2008) and due to less (if any) face‐to‐face contact, qualitative data typically obtained from behavioural observations during testing is scarcer (Schlegel & Gilliland, 2007).

Many medical disciplines have increased accessibility by incorporating telehealth and telemedicine into practice (du Toit et al., 2019). In the last two decades, the availability and acceptance of telemedicine have grown exponentially, in part due to technological advancements and greater availability (Kahn et al., 2016), as well as improved reimbursement and recognition by insurance companies (Yang, 2016; Manchanda, 2020). The advent of COVID‐19 highlighted the need for telehealth services (Monaghesh & Hajizadeh, 2020), and researchers have argued that telehealth services must continue even beyond the pandemic (Thomas et al., 2020). To meet this need, a considerable increase in the availability of telehealth options and online assessment is needed within the field of neuropsychology (Hewitt & Loring, 2020). In the past several years, research has shown that tele‐neuropsychology is a viable and valid method for the assessment of various populations (Brown & Zakzanis, 2023; Galusha‐Glasscock, et al., 2016; Segura & Pompéia, 2021; Sumpter, et al., 2023; Salinas, et al., 2020). However, there is a dearth of literature about assessments specifically designed to be delivered via an online platform, highlighting the need for the development of more tele‐neuropsychology measures, including online assessments (Hewitt & Loring, 2020).

Executive functioning in particular can be difficult to measure using online neuropsychological assessment (Koziol, 2014). In an examination of the literature, Howieson (2019) argues that certain areas of cognition are underrepresented in available assessments, including high‐level cognitive skills, which can include executive functions such as attention, judgement, and decision‐making. Additionally, there is a lack of online‐specific assessments of executive functioning, fine‐motor speed, and dexterity (Peterson et al., 2020). Steps are being taken to fill this gap. In recent years, Survey for Memory, Attention, and Reaction Time (SMART) has been introduced as a self‐administered, web‐based assessment of visual memory, attention/processing speed, and executive functioning using existing tasks within the public domain (Leese et al., 2023). Preliminary research suggests it is a feasible, reliable, and valid method of assessing cognitive performance (Leese et al., 2023), demonstrating the potential of online assessment in assessing executive function. In addition to standard computerized tests, the possibility of using virtual reality (VR) tasks to assess executive function is being explored (Parsons, Courtney, Arizmendi & Dawson, 2011). A recent meta‐analysis describes several VR assessments of executive function created over the past decade where the results of said assessments have shown significant correlations with the results of established measures such as the Stroop test and trail making tests (Pieri et al., 2023).

Available and well‐studied computerized assessments include stand‐alone measures of attention/concentration and executive functioning such as Continuous Performance Tests (CPT) and the Test of Variables of Attention (TOVA), as well as components of cognitive batteries such as Cambridge Neuropsychological Test Automated Battery CANTAB (Kane & Kay, 1992; Robbins et al., 1994). If no appropriate tele‐neuropsychology measure can be obtained, the patient must either attend an in‐person assessment session or the neuropsychologist may forgo these important facets of the neuropsychological battery.

Online and computerized neuropsychological assessments that offer ease of access and administration (both online and in clinical settings) and are easily repeatable are rare but are necessary to improve the measurement of cognitive abilities and to widen access to services provided by the clinical neuropsychologist. To our knowledge, there are few tasks designed to measure processing speed, executive functioning, and simple attention in both an online and computerized fashion. An online assessment is meant to be administered over the internet, whereas a computerized test is a method of administering tests where responses can be recorded and/or assessed electronically. Computerized data collection with continuous variables will enhance the detection of impairments when compared to error tallying approaches that can often be subjective and prone to low inter‐rater reliability. The development of the KS may help mitigate these issues and add value to the neuropsychological test batteries currently in circulation.

The KeyStrokes (KS) test was developed to provide an online and computerized assessment of simple attention, processing speed, and executive functioning. The test requires the use of a standard keyboard and a working internet connection. Like other neuropsychological assessments in its class, the KS test begins with relatively simple tasks that increase in difficulty as the individual progresses through the assessment. It is non‐invasive and requires approximately 10–15 minutes to complete. The KS is based on well‐established tests including the flanker task (Eriksen, 1995), various Stroop type tasks (Golden, 1978; Reynolds & Kamphaus, 2015), the Developmental Neuropsychological Assessment (NEPSY‐II) Inhibition subtest (Korkman et al., 2007), and the Conner's Continuous Performance Test II (CPT II) (Conners, 1992).

An IRB‐approved feasibility study to show proof of concept and establish reliability data for an adult population on the KS test had been previously completed (Lopez et. al., 2024). A total of 1317 participants recruited from Mechanical Turk were given the KS test and included in the analysis. Correlations from subtest to subtest (e.g. Test 1 correlated with Test 2, etc.) were positive, strong (all above *ρ* > .70) and significant (*p* < .001) and as each task increased in difficulty and changed in function (from speeded test to inhibition to inhibition/switching), the correlation declined. The internal consistency of the entire KS test as measured by Cronbach's alpha was excellent (*α* = .95). Regression results were surprising, with age correlating to only two of the seven subtests (Response Speed–Arrows and Inhibition–Words), while education contributed to all subtests. However, this study ultimately had several key limitations, the most prominent being that the KS was based on multiple existing measures of processing speed, attention, and executive functioning, many of which are paper and pencil‐based tasks that cannot be administered online. As such, we were unable to collect data to assess convergent or divergent validity because the feasibility study was conducted during COVID‐19 shutdowns. The aims for this study were to follow up on the initial feasibility study, to continue to show proof of concept, present convergent and divergent validity data, correlational data, and conduct regression analyses for the test.

This pilot study focuses on the KS test's performance as a computerized assessment rather than an online assessment. The primary objective is to assess the correlations between performance on KS subtests and other established neuropsychological assessments, and the secondary objective is to explore whether demographic characteristics such as age and education are predictive or correlative with subtest results.

## METHODS

### Study population

We used a convenience sampling strategy to recruit a total of 87 participants for the current study from a clinical sample of patients referred by their physicians for standard neuropsychological evaluation for various indications. Participants were required to speak English, to be over the age of 18, and not to have severe tremor or a diagnosis of severe mental illness (e.g., schizophrenia). All individuals who met this inclusion criteria were asked to participate in this study. Not all individuals opted in for various reasons (e.g., fatigue, uninterested in participating, etc.).

### Ethical approval and software availability

Participants were presented with an IRB‐approved consent form and HIPAA form that outlined the nature of the study. Informed consent, which included information about the ability to withdraw from the study at any time, was required from the participant before they began the test. Data was keyed anonymously and stored on a secured network drive. The study was approved by the university's Institutional Review Board (Protocol #2448).

The KeyStrokes proprietary software was used for this study. The KeyStrokes software is not currently available to the public; however, the intention is for it to be made open source in the future. The software can be made available for reproducibility upon request.

### Measures

#### The KeyStrokes test

The version of the KS administered for this study has six conditions: two response time trials (arrows, words), three inhibition trials (arrows, words, arrows/words), and one inhibition/switching trial (arrows/words). Compared to the feasibility study, a third response time trial (arrows/words) was removed due to redundancy in tests. We estimated that the entire KS test would take an additional 10 to 15 minutes of time following the patients' standard visit.

Data collection was conducted in two separate clinics using the same protocol. The study took place in a clinic room using a desktop computer with a standard keyboard, specifically a Dell KB500 keyboard and a Dell 27″ monitor. All participants used the same model keyboard and monitor, with similar room illumination and distance to the monitor. Keyboard placement was left to the choice and comfort of the participant as long as the keyboard remained on the desk in front of them.

Participants were provided detailed instructions for completing the task. To proceed with each subtest, the participant needed to correctly answer five items in a row during an example trial. If the participant did not correctly answer five items, the example trial continued until five consecutive items were answered correctly. Feedback was provided to the participant by the software (e.g. ‘That's not quite right. Try again. Press the key you see displayed on the screen’), in addition to a green check mark indicating a correct answer or a red ‘x’ indicating an incorrect answer. The examiner provided feedback to the participant during the example trial only. The 60″ timed trial began after the participant completed an example trial and pushed the spacebar to start.

The order of each item (up, down, left, or right arrow or its corresponding word) was randomized for each participant in each trial and example trial. Data collected included the number of items correct and accuracy. Only the number of items correct was utilized for the purposes of this study.

**FIGURE 1:**
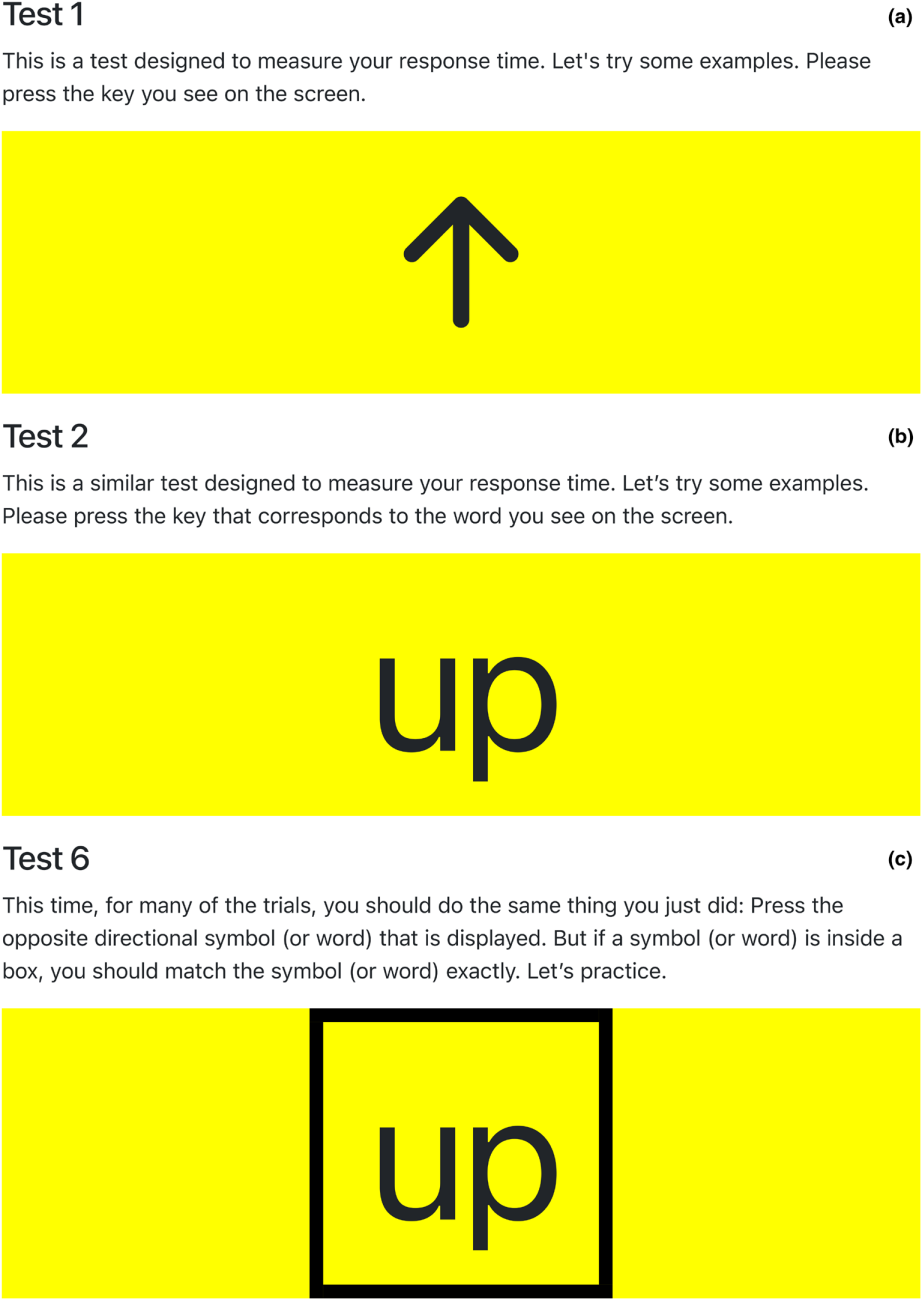
Images from the KeyStrokes test instructions.

The order of administration of trials was the same for each participant, and the instructions were both received through the program and given orally by the testing neuropsychologist. The order and instructions for each trial were as follows:

1. Response Time Trials

- Test 1 (Arrows)

- Participants are shown pictures of arrows (Figure 1a) and asked to press the matching arrow key on the keyboard that they see on the screen as quickly as possible. For example, ‘**↑**’ would indicate to the participant that they would need to press the ‘**↑**’ key on the keyboard. Instructions provided for this test state, ‘This is a test designed to measure your response time. Let's try some examples. Please press the key you see on the screen’. Following the correct completion of the example trials, participants are provided a new prompt to continue the test which states, ‘Good! **↑ ↓ ← →** are the only symbols you will see on this test. When ready, you are to press the key you see on the screen. Work as quickly as you can. You will have 60 s to perform the trial. Try not to make mistakes. Hit “spacebar” when ready’.

ii Test 2 (Words)

- Participants are shown words (Figure 1b) and asked to press the corresponding arrow key on the keyboard that matches the word on the screen. For example, the word ‘up’ would indicate to the participant that they would need to press the ‘**↑**’ key. Similar instructions are provided for this test. For the example trial, instructions read, ‘This is a similar test designed to measure your response time. Let's try some examples. Please press the key that corresponds to the word you see on the screen’. Following the correct completion of the example trials, participants see a new prompt that states, ‘Good! Up, Down, Left, Right are the only words you will see on this test. When ready, you are to press the key that corresponds to the word you see on the screen. Work as quickly as you can. You will have 60 s to perform the trial. Try not to make mistakes. Hit ‘spacebar’ when ready’.

2 Inhibition Trials

- Test 3 (Arrows)

- Like Test 1, participants are shown pictures of arrows; however, they are now instructed to press the opposite arrow key as quickly as possible. For example, ‘**↑**’ would indicate to the participant they would need to press the ‘**↓**’ key on the keyboard. Example instructions state, ‘This test is like the one you just finished, but slightly different. You are to press the opposite directional key than the one you see, ignoring the key that is on the screen. Let's try some examples’. Following correct completion of the example trial, participants are shown the following instruction, ‘Good! **↑ ↓ ← →** are the only symbols you will see on this test. When ready, you are to press the opposite directional key than the one you see, ignoring the key that is on the screen. Work as quickly as you can. You will have 60 s to perform the trial. Try not to make mistakes. Hit ‘spacebar’ when ready’.

ii Test 4 (Words)

- Like Test 2, participants are shown words; however, they are now instructed to press the opposite arrow key on the keyboard than the word displayed on the screen. For example, the word ‘up’ would indicate to the participant that they would need to press the ‘**↓**’ key on the keyboard. Examples note, ‘This test is like the one you just finished, but slightly different. You are to press the opposite directional key than the word you see on the screen. Let's do some examples’. After correctly finishing the example problems, participants see the following instruction, ‘Good! Up, Down, Left, and Right are the only words you will see on this test. When ready, you are to press the opposite directional key that corresponds to the word you see flash on the screen. Work as quickly as you can. You will have 60 s to perform the trial. Try not to make mistakes. Hit “spacebar” when ready’.

iii Test 5 (Arrows/Words combined)

- This test is a mixed combination of both Test 3 and Test 4. Participants are shown either a picture of an arrow or a word and are instructed to press the opposite arrow key on the keyboard than the word or arrow key displayed on the screen. Example instructions stated, ‘This test is just like the two you just completed, but this time you will see both symbols and words. You are to press the opposite directional key or key corresponding to a word than the one you see flash on the screen. Let's do some examples’. After successfully completing the example, instructions stated, ‘Good! Up, Down, Left, Right, **↑ ↓ ← →** are the only words or symbols you will see on this test. When ready, you are to press the opposite directional key or key corresponding to a word than the one you see on the screen. Work as quickly as you can. You will have 60 s to perform the trial. Try not to make mistakes. Hit “spacebar” when ready’.

3 Inhibition/Switching Trials

iv Test 6 (Arrows/Words combined)

- Like Test 5, participants are shown either a picture of an arrow or a word and are instructed to press the opposite arrow key on the keyboard than the word or arrow key displayed on the screen. However, if a word or arrow key is inside a large black box, the participant is asked to press that exact key on the keyboard. See Figure 1c for a visual representation of this task. Example instructions stated, ‘This time, for many of the trials, you should do the same thing you just did: Press the opposite directional symbol (or word) that is displayed. But if a symbol (or word) is inside a box, you should match the symbol (or word) exactly. Let's practice’. After successful completion of the example, participants are shown the following instruction, ‘Good! Up, Down, Left, Right, **↑ ↓ ← →** are the only words or symbols you will see on this test. When ready, you are to press the opposite directional key or key corresponding to a word or match the symbol/word exactly if it is in a box. Work as quickly as you can. You will have 60 s to perform the trial. Try not to make mistakes. Hit “spacebar” when ready’.

#### Additional measures

We also administered a demographics questionnaire about participants' age, ethnicity, education, sexual orientation, and employment history and the following neurocognitive tests: Wechsler Adult intelligence Scale–Fourth Edition (WAIS‐IV), including the digit span (DS; measuring simple attention and working memory), symbol search (SS; measuring processing speed), coding (CD; measuring processing speed), matrix reasoning (MR; measuring visual abstract reasoning), and reliable digit span (RDS) subtests (Wechsler, 2008); the Reynolds Interference Task (Reynolds & Kamphaus, 2015; measuring general neuropsychological integrity); the Trail Making Test part A (TMT‐A; measuring processing speed) and part B (TMT‐B; measuring set‐shifting), (Tombaugh, 2004); the Grooved Pegboard Test (GPT; measuring fine‐motor functioning), (Trites, 1989); the Beck Anxiety Inventory (BAI), (Beck & Steer, 1993); and the Beck Depression Inventory (BDI‐II), (Beck et al., 1996).

Patients were also given tests related to their referral question that were necessary for a complete neuropsychological evaluation and that were not assessed as part of this study.

### Statistical analysis

We used Microsoft Excel® with the Analysis ToolPak and SPSS Statistics to analyse the data collected. The demographic characteristics were analysed using descriptive statistics to obtain frequencies and means. We examined the relationship between various scores on standard neuropsychological tests, demographic information (i.e., age, gender, education, and ethnicity), and the scores on each of the KS subtests using Pearson correlation coefficients. We used Cronbach's alpha to determine the internal consistency reliability of the entire KS test as well as the internal consistency reliability of similar conditions (k1 and k2 (speeded conditions); k3, k4, and k5 (inhibition conditions); and k5 and k6 (inhibition/switching condition), as these are most similar to each other (Cronbach, 1951)). We conducted multiple regression analyses to assess the relationship between each participant's score and their age and education. All the data were assessed at the *p* = .01 and the *p* = .05 level for significance.

### Sensitivity power analysis

To analyse our results to varying effect sizes, a sensitivity power analysis was conducted using SPSS Statistics software. We examined the power of our study across a range of effect sizes including small (Pearson's *r* = .1), medium (Pearson's *r* = .3) and large (Pearson's *r* = .05), while holding the alpha level at .05.

## RESULTS

### Demographics

A mixed clinical sample of 87 individuals chose to participate in the study. The sample was limited to the United States and consisted of participants with an age range of 18–85 (*M* = 54.09, *SD* = 16.24). The population included a total of 49 females and 38 males. Of the participants, 60 self‐identified as Caucasian, 12 as Latino/a, eight as Asian American, three as African American, two as Hawaiian/Pacific Islander, and two as Middle Eastern. The participants were well educated, with all the sample reporting at least a high school diploma. Full demographic information is available in Table 1 Figure 1.

**TABLE 1:** Categorical variable demographic analysis.

	*N*	*M*	*SD*	min.	max.
Age	87	54.09	16.24	18	85

	*N*	Percent
Gender		
Male	38	43.70%
Female	49	56.30%
Ethnicity		
Non‐Hispanic Caucasian, White	60	69.00%
Hispanic, Latino/e	12	13.80%
Asian, Asian American	8	9.20%
Black, African American	3	3.40%
Hawaiian, Pacific Islander	2	2.30%
Middle Eastern	2	2.30%
Level of Education		
12 years	11	12.60%
13 years	9	10.30%
14 years	8	9.20%
15 years	4	4.60%
16 years	27	31.00%
18 years	19	21.80%
19 years	4	4.60%
20 years	5	5.70%

The study population included multiple diagnostic categories including mild cognitive impairment (*n* = 9), traumatic brain injury (*n* = 19), multiple sclerosis (*n* = 4), worried well (i.e. cognitively normal; *n* = 29), and other varying diagnoses (*n* = 26), many comorbid, including but not limited to alcohol use disorder, generalized anxiety disorder, normal pressure hydrocephalus, Parkinson's disease, COVID‐19, and attention deficit hyperactivity disorder (ADHD).

### Sensitivity power analysis

A sensitivity power analysis indicated that a sample size of 29 participants would be required to detect a large‐sized Pearson correlation (*r* = .5) with 80% power at a significance level of .05, a sample size of 84 participants to detect a medium‐sized Pearson correlation (*r* = .3), and a sample size of 782 to detect a small‐sized Pearson correlation (*r* = .1). In the current study, for a small effect size (Pearson's *r* = .1), the sample of 87 participants showed a power of 1‐*β* = .15. For a medium effect size (Pearson's *r* = .3), the sample of 87 participants showed a power of 1‐*β* = .81. For a large effect size (Pearson's *r* = .5), the sample of 87 participants showed a power of 1‐*β* = .99.

### Internal consistency

The internal consistency of the entire KS test was excellent (*α* = .96). This was similar to the feasibility study which showed that the entire KS test has an internal consistency of *α* = .95. Internal consistency was excellent for speeded conditions (k1 and k2), (*α* = .91), inhibition conditions (k3, k4 and k5), (*α* = .93), and between the inhibition/switching condition (k6) and the most similar inhibition condition (k5), (*α* = .91). We used internal consistency to highlight the consistency of measurement across similar subtests and the entire KS test.

### Correlation analyses

Correlations between the six subtests were computed. All correlations were positive, strong (above *ρ* > .72), and significant (*p* < .001), (Table 2). We observed positive correlations between speeded conditions (k1 and k2), inhibition conditions (k3, k4, and k5), and the inhibition/switching condition (k5 and k6). The feasibility study showed similar results, with positive, strong (all above *ρ* > .70), and significant (*p* < .001) correlations between the six subtests.

**TABLE 2:** KS subtest correlations.

Correlations		K1	K2	K3	K4	K5	K6
K1	Pearson Correlation		.89**	.87**	.72**	.79**	.80**
	Sig. (2‐tailed)		<.001	<.001	<.001	<.001	<.001
	*N*		87	87	87	87	87
K2	Pearson Correlation	.89**		.87**	.81**	.81**	.77**
	Sig. (2‐tailed)	<.001		<.001	<.001	<.001	<.001
	*N*	87		87	87	87	87
K3	Pearson Correlation	.87**	.87**		.79**	.87**	.76**
	Sig. (2‐tailed)	<.001	<.001		<.001	<.001	<.001
	*N*	87	87		87	87	87
K4	Pearson Correlation	.72**	.81**	.79**		.88**	.82**
	Sig. (2‐tailed)	<.001	<.001	<.001		<.001	<.001
	*N*	87	87	87		87	87
K5	Pearson Correlation	.79**	.81**	.87**	.88**		.84**
	Sig. (2‐tailed)	<.001	<.001	<.001	<.001		<.001
	*N*	87	87	87	87		87
K6	Pearson Correlation	.80**	.77**	.76**	.82**	.84**	
	Sig. (2‐tailed)	<.001	<.001	<.001	<.001	<.001	
	*N*	87	87	87	87	87	

**Correlation is significant at the .01 level (2‐tailed).

Correlations were also computed between each of the six subtests and multiple neurocognitive tests, including the WAIS‐IV subtests of digit span (DS), symbol search (SS), coding (CD), matrix reasoning (MR) and reliable digit span (RDS); trail making tests A and B (TMT‐A and TMT‐B); the Reynolds Interference Test subtests of colour interference (CI) and object interference (OI); grooved pegboard test (GPT); the Beck anxiety inventory (BAI); and the Beck depression inventory (BDI‐II).

Subtest one (k1) showed a positive correlation with DS, SS, CD, MR, OI, and CI and a negative correlation with TMT‐A, TMT‐B and the GPT. There was not a significant correlation between k1 and RDS, the BAI, or the BDI‐II. Subtest two (k2) showed a positive correlation with DS, SS, CD, MR, RDS, OI and CI and a negative correlation with TMT‐A, TMT‐B, and the GPT. There were no significant correlations between k2 and the BAI or the BDI‐II. Subtest three (k3) showed a positive correlation with DS, SS, CD, MR, OI and CI and a negative correlation with TMT‐A, TMT‐B and the GPT. There was not a significant correlation between k3 and RDS, the BAI, or the BDI‐II. Subtest four (k4) showed a positive correlation with DS, SS, CD, MR, RDS, OI, and CI and a negative correlation with TMT‐B and the GPT. There was not a significant correlation between k4 and TMT‐A, the BAI, or the BDI‐II. Subtest five (k5) showed a positive correlation with DS, SS, CD, MR, OI, and CI and a negative correlation with TMT‐A, TMT‐B, and the GPT. There was not a significant correlation between k5, RDS, the BAI, or the BDI‐II. Subtest six (k6) showed a positive correlation with SS, CD, MR, OI and CI and a negative correlation with TMT‐A, TMT‐B, and the GPT. There was not a significant correlation between k6, DS, RDS, the BAI, or the BDI‐II. See Table 3 for more information.

**TABLE 3:** KS subtest correlations with other neuropsychological measures.

	WAIS‐IV					TMT		RIT		GPT	BDI‐II	BAI
	DS	SS	CD	MR	RDS	TMT‐A	TMT‐B	OI	CI			
	*r*(86)	*r*(84)	*r*(84)	*r*(72)	*r*(85)	*r*(86)	*r*(84)	*r*(44)	*r*(44)	*r*(74)	*r*(60)	*r*(59)
	*p*‐value (*r*)	*p*‐value (*r*)	*p*‐value (*r*)	*p*‐value (*r*)	*p*‐value (*r*)	*p*‐value (*r*)	*p*‐value (*r*)	*p*‐value (*r*)	*p*‐value (*r*)	*p*‐value (*r*)	*p*‐value (*r*)	*p*‐value (*r*)
K1	.28	.57	.65	.46	.16	−.31	−.46	.54	.64	−.64	.01	−.10
	.010*	<.001**	<.001**	<.001**	.141	.004**	<.001**	<.001**	<.001**	<.001**	.923	.433
K2	.39	.53	.63	.47	.29	−.26	−.53	.60	.74	−.65	.05	−.08
	<.001**	<.001**	<.001**	<.001**	.006**	.017*	<.001**	<.001**	<.001**	<.001**	.692	.529
K3	.32	.65	.73	.53	.20	−.34	−.56	.48	.65	−.67	.08	−.14
	.002**	<.001**	<.001**	<.001**	.060	.001**	<.001**	.001**	<.001**	<.001**	.552	.302
K4	.35	.53	.67	.43	.27	−.17	−.57	.56	.70	−.57	.08	−.16
	<.001**	.001**	<.001**	<.001**	.013*	.122	<.001**	<.001**	<.001**	<.001**	.538	.228
K5	.30	.59	.69	.52	.20	−.24	−.63	.45	.60	−.57	.07	−.24
	.005**	<.001**	<.001**	<.001**	.064	.025*	<.001**	.002**	<.001**	<.001**	.608	.064
K6	.21	.52	.65	.51	.14	−.23	−.54	.55	.66	−.54	.06	−.13
	.051	<.001**	<.001**	<.001**	.205	.035*	<.001**	<.001**	<.001**	<.001**	.643	.311

*Correlation is significant at the .05 level (2‐tailed).

**Correlation is significant at the .01 level (2‐tailed).

We also computed correlations between each of the six subtests and demographic characteristics (i.e., age, education, gender, and ethnicity). We observed significant negative correlations between age and performance on all six KS subtests. Significant positive correlations between ethnicity were noted for four of the six KS subtests. No significant correlations were identified between subtest performance and other demographic characteristics (Table 4).

**TABLE 4:** KS subtest correlations with age, education, ethnicity, and gender.

	Age	Education	Ethnicity	Gender
	*r*(87)	*r*(87)	*r*(87)	*r*(87)
	*p*‐value	*p*‐value	*p*‐value	*p*‐value
K1	−.69	.04	.24	.21
	<.001**	.713	.024*	.055
K2	−.54	.12	.15	.12
	<.001**	.251	.166	.266
K3	−.58	.14	.25	.14
	<.001**	.207	.017*	.211
K4	−.45	.15	.22	.13
	<.001**	.171	.044*	.232
K5	−.54	.12	.20	.11
	<.001**	.281	.058	.303
K6	−.61	.12	.23	.10
	<.001**	.273	.034*	.356

*Correlation is significant at the .05 level (2‐tailed).

**Correlation is significant at the .01 level (2‐tailed).

### Multiple regression analysis

We conducted a multiple regression analysis to determine if age and education predicted performance on each of the KS subtests within the constraints of the smaller sample size. For all subtests, age added significantly to the prediction, whereas education did not add significantly to any subtest (Table 5).

**TABLE 5:** Age and education regression.

Subtest	Age	Education	Regression Summary
	*p*‐value	*p*‐value	
K1	<.001	.52	F(2, 84) = 38.78, *p* < .001, R^2^ = .48
K2	<.001	.15	F(2, 84) = 18.66, *p* < .001, R^2^ = .31
K3	<.001	.10	F(2, 84) = 22.89, *p* < .001, R^2^ = .35
K4	<.001	.11	F(2, 84) = 12.19, *p* < .001, R^2^ = .23
K5	<.001	.17	F(2, 84) = 18.64, *p* < .001, R^2^ = .31
K6	<.001	.14	F(2, 84) = 27.05, *p* < .001, R^2^ = .39

## CONCLUSION AND DISCUSSION

The KeyStrokes (KS) test was developed as an online and computerized assessment of simple attention, processing speed, and executive functioning. This project was intended as a follow‐up to the feasibility study to further show proof of concept, assess the correlations between performance on KS subtests and other established neuropsychological assessments, and explore whether demographic characteristics such as age and education are predictive or correlative with results. The KS demonstrated excellent internal consistency as an entire test and between similar conditions (i.e., speeded, inhibition, etc.), as well as showing strong and positive correlations between performance on each KS subtest. It also showed strong correlational data with multiple well‐established neuropsychological tests in a mixed clinical sample.

In contrast to the original feasibility study, correlation analyses showed age correlating with all subtests measured in this study, similar to trends seen in traditional paper and pencil‐based tasks. These results add additional data to the literature showing that age plays a large role in many areas of cognition (Anderson et al., 2004). Education, however, did not correlate with any subtest. Simple tasks are often less impacted by education, which could explain the lack of impact of education on this test (Wartella & Jennings, 2000). However, studies have shown that families with higher incomes and higher education levels are more likely to own computers in childhood and have internet access (Calvert et al., 2005), so it is reasonable to expect a correlation with education among individuals. Given the smaller sample size and relatively equal spread of education levels among the cohort (none of the participants in this study had less than 12 years of education), it is possible that the effect of education on performance is not currently being seen in the data.

Analysis of the correlations between the KS subtests and multiple well‐established neuropsychological tests showed the possible strength of the KS as a potentially new neurocognitive measure, most notably in the areas of attention (DS), processing speed (CD, SS, and TMT‐A), and executive function (OI, CI, and TMT‐B). The positive correlations between DS, CD, and SS align well with total scores across each KS subtest, as higher scores on the KS subtests correlate with higher scores on each WAIS‐IV subtest (Wechsler, 2008). Similarly, higher total scores (i.e., better performance) on the RIT subtests correlate with higher scores on the KS subtests (Reynolds & Kamphaus, 2015). For trail making tests, the negative correlations appear to show accurate measurements as well, since lower scores on trail making tests are a function of better performance (Tombaugh, 2004). The significant correlation between these measures and the KS test provides evidence of criterion validity (Swerdlik & Cohen, 2005). The KS subtests correlating with the MR subtest of the WAIS‐IV is a surprising finding and may be related to the visual nature of the KS itself rather than a measure of visual abstract reasoning (Wechsler, 2008). Reliable digit span (RDS) also appeared to be correlated with two out of the six KS subtests. Traditionally, a lower score on the RDS (often varying with clinical diagnosis) is shown to represent poor engagement (Schroeder et al., 2012). The positive correlations found on KS subtests two and four could suggest that these subtests may be measuring engagement in some way much like the RDS (i.e., higher total scores are indicative of better engagement). Interestingly, these two subtests are ‘words’ only. Additional research would be needed to explore the significance of this finding. Correlations with the GPT provide evidence that the KS, while impactful for measuring certain cognitive areas, may not necessarily be a viable option for individuals with fine‐motor challenges. Still, the utility of the KS as a measure appears to be strong when controlling for fine‐motor impacts. Lastly, the KS does not appear to correlate consistently with mood or anxiety measures, which is expected as the KS is not intended to measure such issues. Analysis of the current findings appears to provide evidence of discriminant validity for the KS (Hubley, 2014). Overall, the strong correlation data between the KS and multiple well‐established neuropsychological tests suggest that further study of the KS as an alternative option to traditional pencil‐based testing is worthwhile. These data show multiple lines of validity evidence which provide relevant information for the intended test score interpretation (American Educational Research Association, 2014).

If further research on the KS determines its validity and reliability, the KS could have a positive impact on neuropsychology and tele‐neuropsychology. First, the KS is designed to be used in multiple settings, including but not limited to inpatient bedside assessment, outpatient clinics, and outside of clinical settings (i.e., home). The ease of use that this assessment method provides may open access to individuals who are unable to easily receive neuropsychological services. Administratively, the KS could be a viable tool in providing a snapshot of cognitive functioning across multiple settings. The built‐in randomness of the test (no two administrations of a test condition are the same) may make it possible to track change across much shorter intervals while mitigating practice effects. As such, it is possible that the KS could be most useful in areas that have previously been out of reach for neuropsychologists and clinicians, including tracking day‐to‐day recovery from traumatic brain injury (even useful as a possible treatment), the impacts of medication use on various cognitive functions (before, during, and/or after prolonged use or effects of medication dosage changes), post‐surgical recovery, or daily to weekly assessment of neurological disease progression. These areas may be particularly interesting for future study.

The KS could provide some other advantages over other tests as well. It was designed to be administered without the use of a proctor, which allows it to be deployed more readily in situations that require remote administration and minimal training on the instrument. Similarly, it could also be a useful tool for neuropsychologists and medical providers who wish to quickly and objectively understand how treatments are impacting their patient's cognition without the need for a formal clinic visit. The creation of the KS is also a push towards the expansion of accessibility in neuropsychology. As we have seen in the last few years, this limitation has greatly impacted the way neuropsychological services are rendered to those who need them most (Harrell et al., 2014). The continued creation of computerized assessments that are accessible to the clinical neuropsychologist and the patients they serve is imperative to promoting growth and innovation within the field. We hope that further research on the KS can help meet the growing clinical need for innovation and assessment in neuropsychology.

Key limitations in our study include the smaller sample size, lack of serial testing, lack of health control, and reliance on normative percentiles. Of note, the sample size may limit the interpretability of the results of this study, particularly the regression analyses. For example, our multiple regression analysis found that age predicted performance, whereas education was not as impactful. It is certainly possible that results may be different within a larger study population as education is often seen as a significant factor in neuropsychological assessment (Rohit et al., 2007). Regarding ethnicity, gender, and education, the relatively smaller sample size also resulted in a predominately Caucasian and female cohort, and a generally restricted range of education, which causes difficulties in both stratifying results based on these three metrics and analysing their impact on test performance. Continued research would do well to include a more diverse and larger cohort.

Test–retest reliability data is also still a necessity, given that the KS test is attempting to measure stability over time and/or change in ability. Test–retest reliability is a more relevant statistic than internal consistency in this instance. While neuropsychological tests have been shown to have generally strong test–retest reliability (Williams et al., 2005), measurement of the construct is still recommended for new tests that are based on existing tests. However, gathering test–retest data was not feasible for this study given the clinical setting in which it was performed. Future studies on the KS focusing on this type of reliability are necessary. Nonetheless, we reported internal consistency to highlight the consistency of measurement across similar subtests and the entire KS test.

The sensitivity power analysis conducted showed that our results had sufficient power to detect a medium effect size. That said, power significantly decreased when looking at smaller effect sizes. Nonetheless, while our findings show significance, interpretation should be made with caution due to the potential for a Type II error in the population should the true effect size be smaller.

The current iteration of the KS test requires the use of a standard keyboard which potentially limits its accessibility. Additionally, the diversity of keyboards available on the market with arrow keys varying in the distance between keys, locations of the keys, and key sensitivity may lead to challenges related to generalizability and speed of completion across keyboard types (Cernich et al., 2007). As previously discussed, the KS may be ineffective in cases where fine‐motor functioning is significantly impacted, a limitation shared among similar motor‐dependent neuropsychological tests. While participants were screened for significant tremor to mitigate this, results proved that fine‐motor dysfunction is an adequate concern. The reliance on normative percentiles introduces the issue of standardization with the KS measure due to ever‐changing task patterns. While this may mitigate a ‘repeat effect’ common to routine neuropsychological examinations, it may also prove to be an additional challenge in norming the KS test in the future.

### Future studies

Repeated study in an outpatient or laboratory setting would provide additional validity and reliability (i.e. test–retest) data to the KS test. Replication studies would also be helpful in determining its viability in multiple settings. Additionally, more study with identified clinical and normal populations would be beneficial because it would allow for an analysis based on diagnostic category to be completed. Lastly, a study that is centered around repeated administrations over shorter time frames (e.g. days to weeks) would be crucial in determining its efficacy in acute clinical settings and its usefulness in tracking change in quick succession (e.g. inpatient admit/discharge decisions). Repeating this study with multiple groups would also help clarify whether the KS is useful for screening for impairment of simple attention, processing speed, or executive function in clinical populations. The KS currently has functionality to measure keystroke rate (i.e. latency) which was not assessed for this study. Future studies on the KS could benefit from analysis of the latency between keystrokes to see the value of assessing this type of metric in clinical settings.

## AUTHOR CONTRIBUTIONS

**Michael Lopez:** Conceptualization; data curation; formal analysis; funding acquisition; investigation; methodology; software; resources; supervision; project administration; writing – original draft; writing – review and editing; visualization; validation. **John Fulton:** Conceptualization; supervision; funding acquisition; formal analysis; resources; data curation; investigation; project administration. **Hayley Kristinsson:** Writing – review and editing; resources; data curation; supervision. **Sahra Kim:** Resources; writing – original draft; formal analysis. **Elizabeth Stuart:** Resources; conceptualization; formal analysis; writing – original draft; data curation. **Patrick Chen:** Resources; writing – review and editing. **Aaron Thomas:** Writing – review and editing; visualization; resources. **Megan Hussey‐Zommers:** Writing – review and editing; visualization; resources. **Rohan Roy:** Software; resources. **Arunima Kapoor:** Writing – review and editing; formal analysis. **Alexis Conrad:** Writing – review and editing; formal analysis.

## CONFLICT OF INTEREST

None of the authors has a conflict of interest to report.

## Data Availability

The data that support the findings of this study are available upon request.
